# Supplementary material for: Uncovering Protein Ensembles: Automated Multiconformer Model Building for X-ray Crystallography and Cryo-EM
Source: bioRxiv. 2024 Apr 6:2023.06.28.546963. Preprint. [Version 3] doi: 10.1101/2023.06.28.546963 (PMC10327213; doi:10.1101/2023.06.28.546963)
Supplement: Supplementary file 1 [file NIHPP2023.06.28.546963V3-supplement-1.pdf]

bioRxiv preprint doi: https://doi.org/10.1101/2023.06.28.546963; this version posted April 6, 2024. The copyright holder for this preprint (which was not certified by peer review) is the author/funder, who has granted bioRxiv a license to display the preprint in perpetuity. It is made available under aCC-BY 4.0 International license.

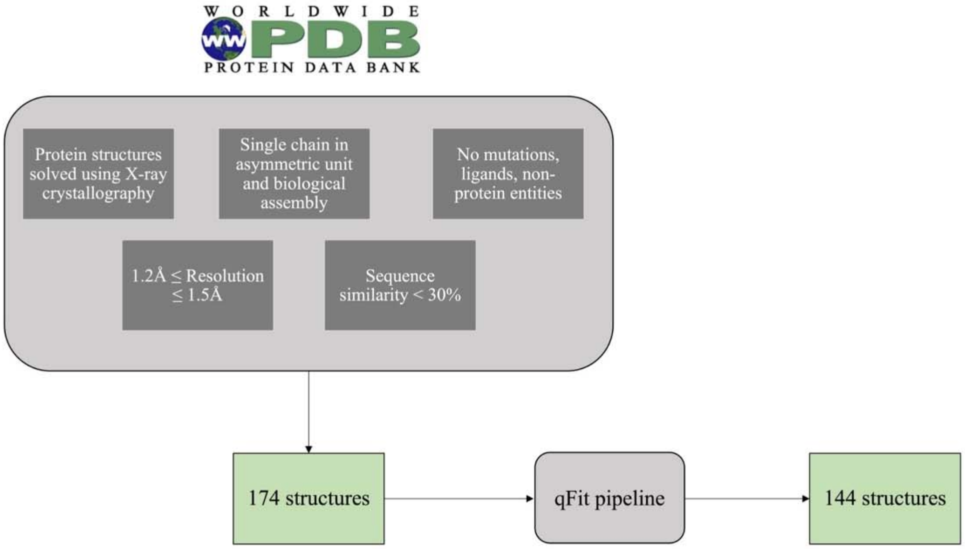

Supplementary Figure 1. Flow diagram of the selection of the test set PDBs.

bioRxiv preprint doi: https://doi.org/10.1101/2023.06.28.546963; this version posted April 6, 2024. The copyright holder for this preprint (which was not certified by peer review) is the author/funder, who has granted bioRxiv a license to display the preprint in perpetuity. It is made available under aCC-BY 4.0 International license.

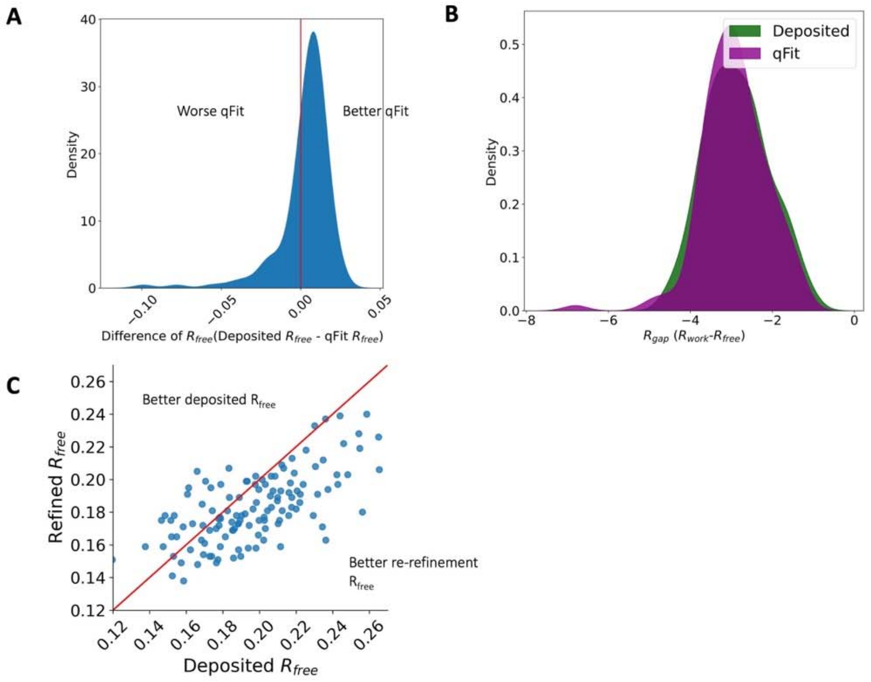

Supplementary Figure 2. R_{free} and R-gap distributions.
A. Distribution of difference of R_{free} between deposited and qFit models. The median difference in R_{free} is 0.6%. Median deposited models R_{free}: 18.1%, median qFit models R_{free}: 17.5%.
B. Distribution of R-gap values between deposited and qFit models (median deposited model: 3.0%, median qFit model: 3.0%).
C. Distribution of R_{free} value in PDB deposited models versus re-refined deposited models. In this manuscript, deposited models refer to the re-refined deposited models.

bioRxiv preprint doi: https://doi.org/10.1101/2023.06.28.546963; this version posted April 6, 2024. The copyright holder for this preprint (which was not certified by peer review) is the author/funder, who has granted bioRxiv a license to display the preprint in perpetuity. It is made available under aCC-BY 4.0 International license.

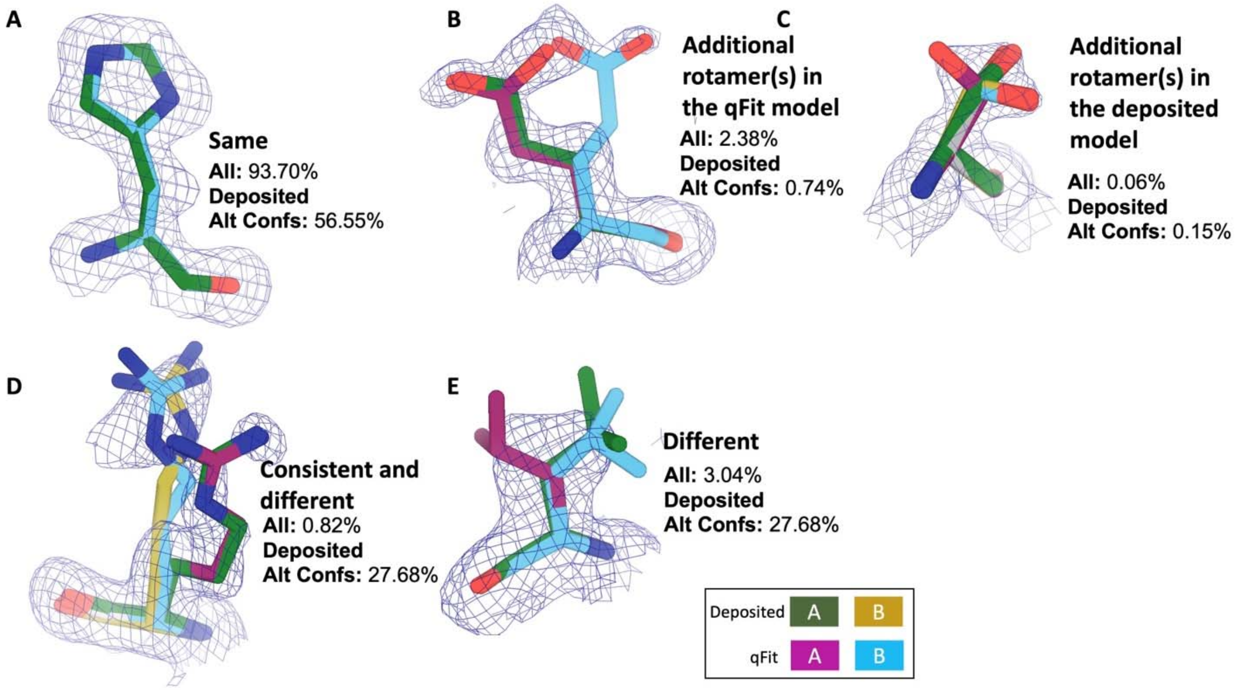

Supplementary Figure 3. Examples of rotamer state categories.
Meshes represent 2Fo-Fc density at 1 σ. Green and yellow sticks represent deposited conformer(s). Blue and magenta sticks represent qFit conformer(s).
A. Same: The entire set of rotamers identified in the deposited and qFit models are the same (PDB: 1BN6, His199).
B. Additional rotamer(s) in the qFit model: Deposited and qFit models share at least one rotamer, and at least one additional rotamer was identified in the qFit model (PDB: 3CX2, Glu165).
C. Additional rotamer(s) in the deposited model: Deposited and qFit models share at least one rotamer, and at least one additional rotamer was identified in the deposited model (PDB: 4P48, Ser6).
D. Consistent and different: Deposited and qFit models share at least one rotamer, and at least one unique additional rotamer was identified in both the deposited model and the qFit model (PDB: 3HP4, Arg81).
E. Different: The rotamers in the deposited and qFit models are all different (PDB: 1BN6, Glu110).

bioRxiv preprint doi: https://doi.org/10.1101/2023.06.28.546963; this version posted April 6, 2024. The copyright holder for this preprint (which was not certified by peer review) is the author/funder, who has granted bioRxiv a license to display the preprint in perpetuity. It is made available under aCC-BY 4.0 International license.

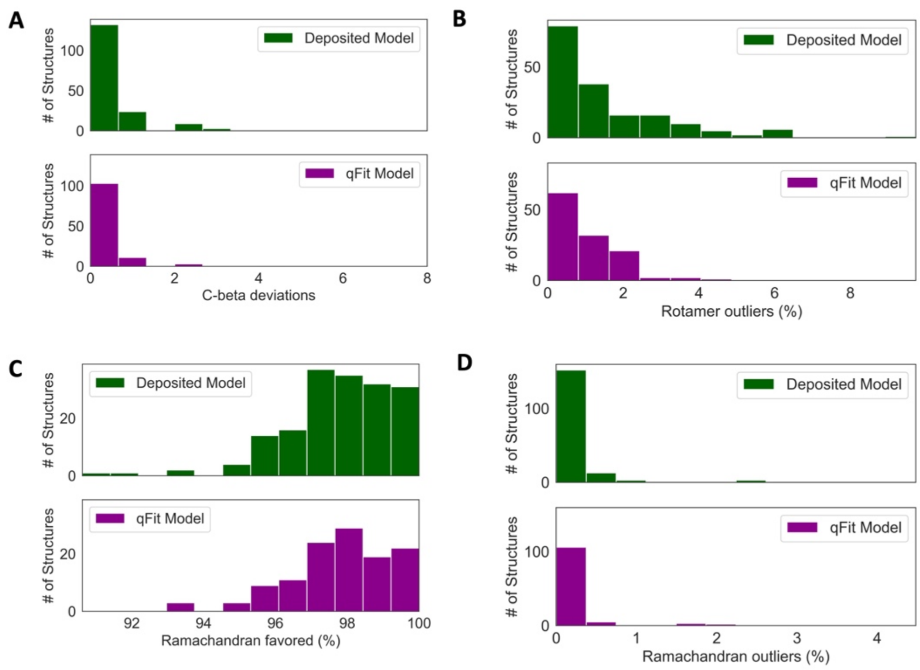

Supplementary Figure 4. Deposited versus qFit model geometry.
A. Count of number of Cβ deviation (>0.25Å) per model (deposited model: 0.0 median [interquartile range: 0.0-0.0], qFit model: 0.0 median [interquartile range: 0.0-0.0]), p-value=0.37 from two-sided t-test.
B. Median count of number of rotamer outliers per model (deposited model: 0.94 [0.00-2.12], qFit model: 0.81 [0.35-1.60]), p-value=0.73 from two-sided t-test.
C. Percent of Ramachandran favored per model: deposited model (97.70 [96.90-98.93], qFit model: 98.0 [97.05-98.97]), p-value=0.77 from two-sided t-test.
D. Percent of Ramachandran outliers per model (deposited model 0.0 [0.0-0.0], qFit model: 0.0 [0.0-0.0)]), p-value=0.57 from two-sided t-test.

bioRxiv preprint doi: https://doi.org/10.1101/2023.06.28.546963; this version posted April 6, 2024. The copyright holder for this preprint (which was not certified by peer review) is the author/funder, who has granted bioRxiv a license to display the preprint in perpetuity. It is made available under aCC-BY 4.0 International license.

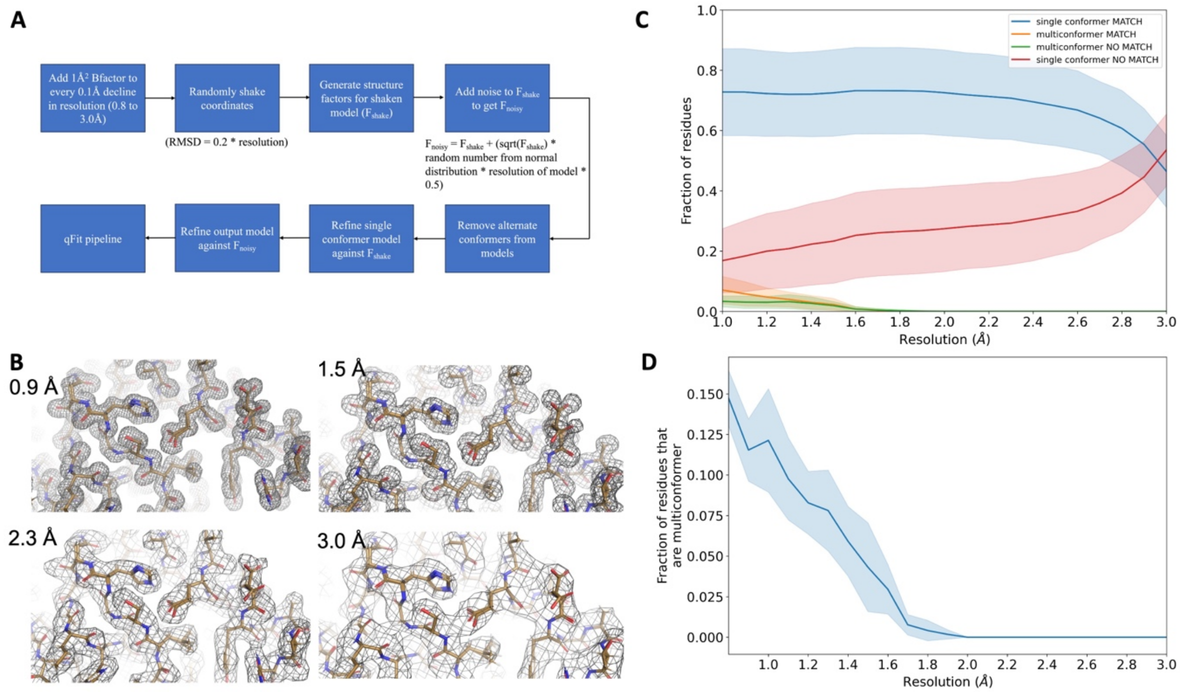

Supplementary Figure 5.

A. Protocol for generating synthetic structure factors at various resolutions starting from the ground truth model. For the 7KR0 dataset, all the steps starting from random shaking of coordinates were done 10 times for each resolution. For the larger test dataset, all steps were only done once.

B. A visualization of synthetic maps generated for the models at varying resolution. The loss in detail of density is clearly visible with worsening resolution.

C. Proportion of all residues in qFit models which have been modeled as multiconformers in the 7KR0 dataset, as a function of resolution. The shaded region around the line indicates the spread across 10 runs at every resolution step.

D. Proportion of all residues in the qFit models of qFit test dataset which are modeled as multiconformer match (orange), single conformer match (blue), multiconformer no match (green), and single conformer no match as a function of resolution of input data. The shaded region around the lines indicates the spread across the qFit test dataset which consists of 103 proteins.

bioRxiv preprint doi: https://doi.org/10.1101/2023.06.28.546963; this version posted April 6, 2024. The copyright holder for this preprint (which was not certified by peer review) is the author/funder, who has granted bioRxiv a license to display the preprint in perpetuity. It is made available under aCC-BY 4.0 International license.

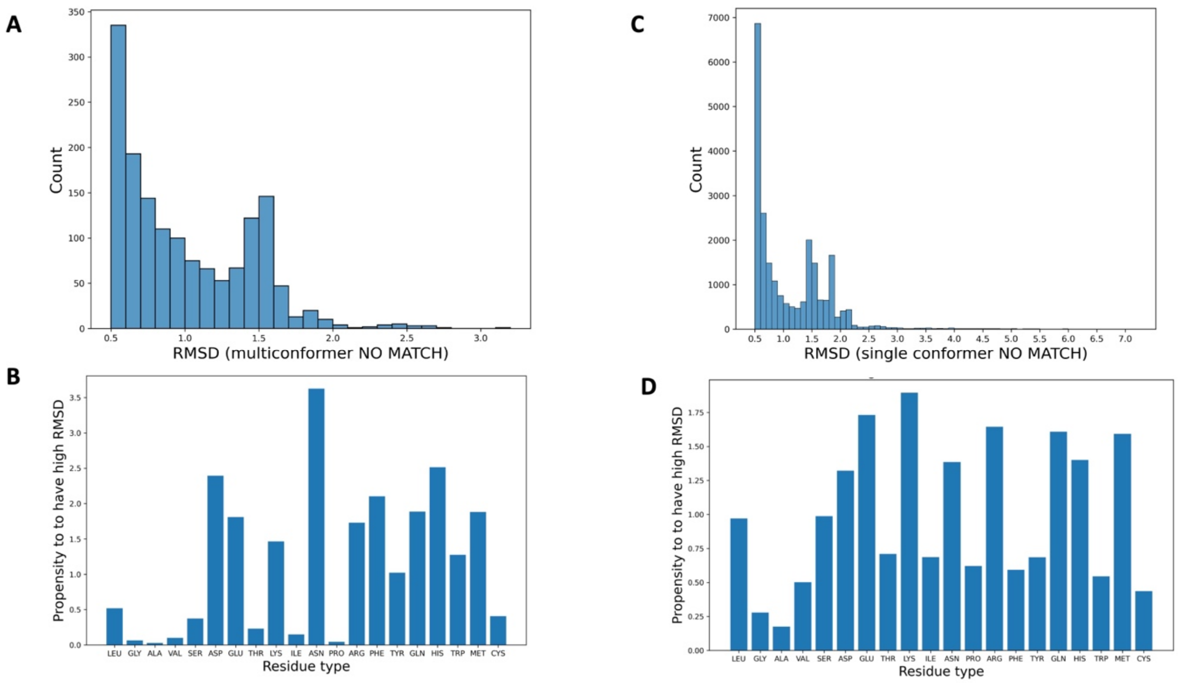

Supplementary Figure 6.

A. The distribution of RMSD between qFit residues and corresponding ground truth residues (qFit test set) whenever the RMSD is higher than the 0.5 Å cutoff, resulting in the qFit residues being classified as multiconformer no match.

B. The propensity of each residue type to be modeled with high RMSD from the ground truth (qFit test set), resulting in being classified as multiconformer no match. This propensity of a residue type x is calculated as the ratio between (i) proportion of residue type x among all the residues with a high RMSD and (ii) proportion of residue type x in the entire dataset.

C. The distribution of RMSD between qFit residues and corresponding ground truth residues (qFit test set) whenever the RMSD is higher than the 0.5 Å cutoff, resulting in the qFit residues being classified as single conformer no match.

D. The propensity of each residue type to be modeled with high RMSD from the ground truth (qFit test set), resulting in being classified as single conformer no match.

bioRxiv preprint doi: https://doi.org/10.1101/2023.06.28.546963; this version posted April 6, 2024. The copyright holder for this preprint (which was not certified by peer review) is the author/funder, who has granted bioRxiv a license to display the preprint in perpetuity. It is made available under aCC-BY 4.0 International license.

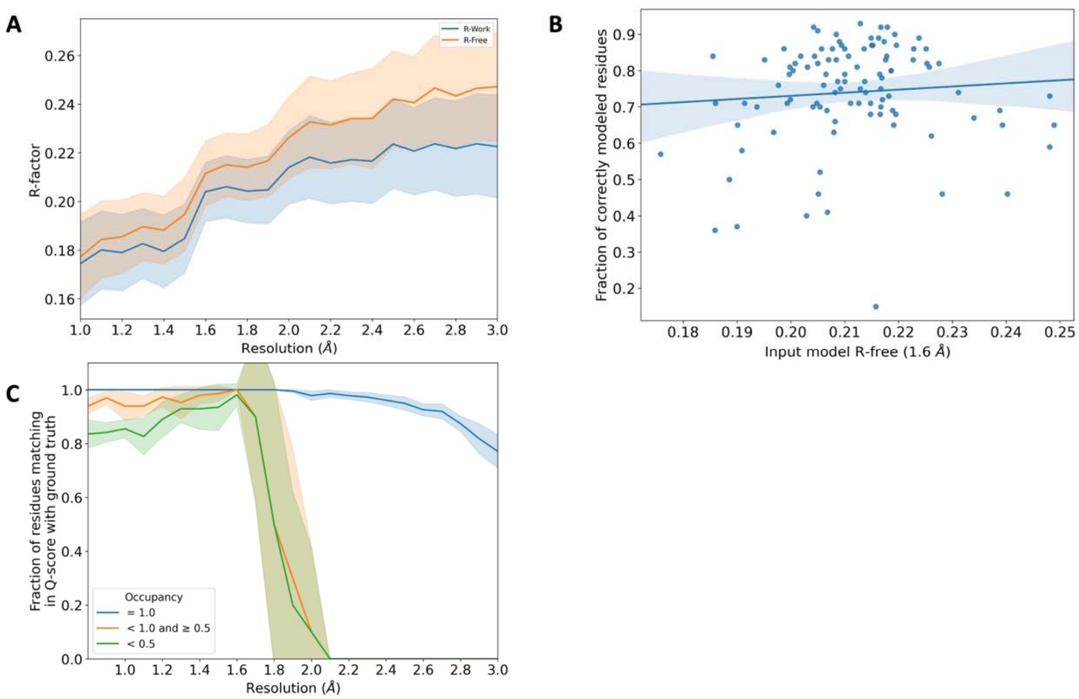

Supplementary Figure 7.

A. R_{work} (blue) and R_{free} (orange) distribution of the input model from the qFit test dataset. These correspond to the models obtained after refining against F_{noisy} structure factors (see **Supplementary Figure 5A**). The shaded region around the lines indicates the spread (standard deviation) across the qFit test dataset.

B. Fraction of correctly modeled qFit residues (match multiconformer + match single conformer) as a function of input model R_{free} for all structures in the qFit test dataset at 1.6 Å resolution (input R_{free} range: 0.17 to 0.25, n=103). The shaded region denotes the 95% confidence interval.

C. The fraction of residues in the qFit models of the 7KR0 dataset with a Q-score within 0.01 of that of the ground truth model as a function of resolution. In multiconformer residues, Q-score for every alternate conformer is calculated separately. Q-scores of residues (or) conformations which have matching occupancy (range) are compared. Occupancy of conformations were binned into three classes – occupancy equal to 1 (blue), 1 > occupancy >= 0.5 (orange) and occupancy < 0.5 (green).

bioRxiv preprint doi: https://doi.org/10.1101/2023.06.28.546963; this version posted April 6, 2024. The copyright holder for this preprint (which was not certified by peer review) is the author/funder, who has granted bioRxiv a license to display the preprint in perpetuity. It is made available under aCC-BY 4.0 International license.

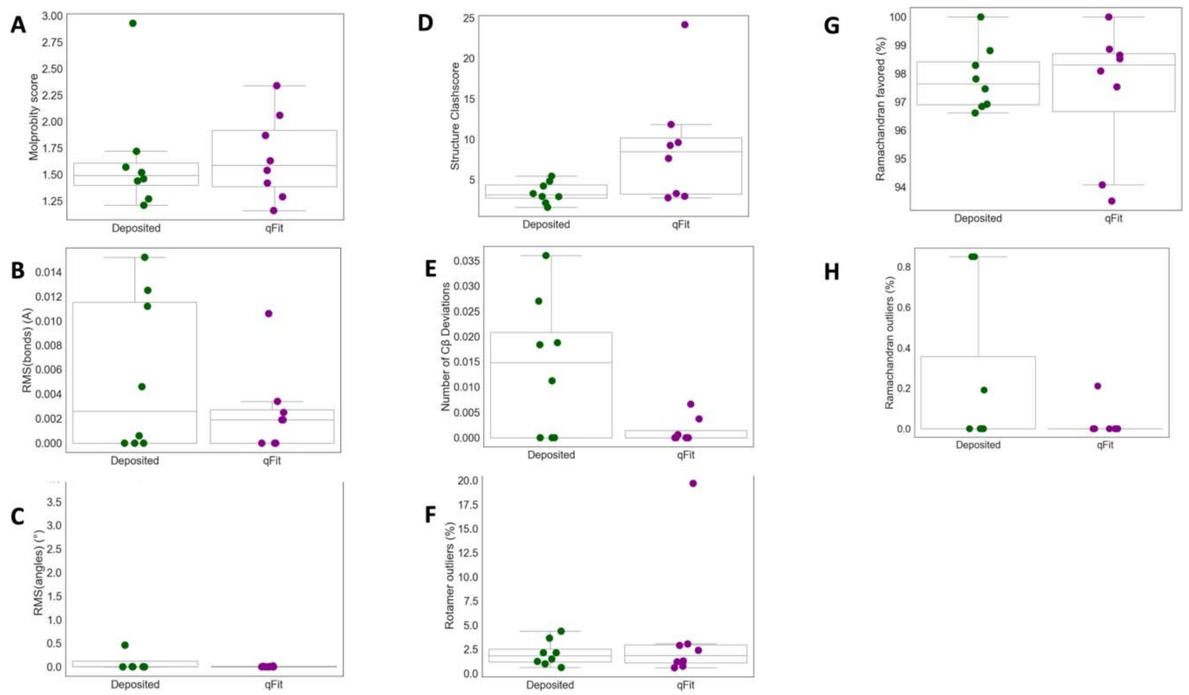

Supplementary Figure 8.

A. MolProbity score(deposited model: 1.49 (median) [1.40-1.61] (interquartile range), qFit model: 1.59 (median) [1.39-1.92] (interquartile range))

B. Model average of RMSD of model bond length from idealized bond length(Å)(deposited model: 0.00 [0.00-0.01], qFit model: 0.00 [0.00-0.00])

C.Model average of RMSD of model bond angle from idealized bond angle(Å)(deposited model: 0.00 [0.00-0.11], qFit model: 0.00 [0.00-0.01])

D. Number of residues with clashscore(deposited model: 3.15 [2.74-4.39], qFit model: 8.45 [3.22-10.17])

E. Number of Cβ deviation (>0.25 Å) per model (deposited model: 0.02 [0.00-0.02], qFit model: 0.00 [0.00-0.00])

F. Number of rotamer outliers per model(deposited model: 2.0 [2.0-2.0], qFit model: 2.0 [1.0-3.0]**)**

G. Percent of Ramachandran favored per model(deposited model: 97.6 [96.9-98.9], qFit model: 98.3 [96.7-98.7)])

H. Percent of Ramachandran outliers per model (deposited model: 0.0 [0.0-0.0], qFit model: 0.0 [0.0-0.0])
